# Epicatechin‐mediated modulation of the Nrf2/HO‐1 pathway alleviates senile cerebral ischemic/reperfusion injury

**DOI:** 10.1002/fsn3.4253

**Published:** 2024-06-21

**Authors:** Changyue Jiang, Xiangzhen Zhuge, Deli Li, Menghua Chen, Wanxiang Hu, Lu Xie

**Affiliations:** ^1^ Department of Physiology Guangxi Medical University Nanning China; ^2^ HIV/AIDS Clinical Treatment Center of Guangxi (Nanning) and The Fourth Hospital of Nanning Nanning China; ^3^ Department of Cardiology Foresea Life Insurance Nanning Hospital Nanning China

**Keywords:** aging, antioxidants, cerebral ischemia–reperfusion injury, Nrf2/ARE, oxidative stress

## Abstract

Excessive reactive oxygen species (ROS) generated during cerebral ischemic reperfusion (CIRI) are crucial for subsequent tissue damage. However, despite the potential benefits of antioxidants reported in clinical applications, few have proven effective in treating CIRI, particularly in the elderly. Epicatechin (EC) is a catechol flavonoid monomer derived from natural tea plants. Multiple phenolic hydroxyl groups give it strong antioxidant properties, which can not only degrade ROS through chemical reactions between hydroxyl and ROS but also enhance the activity of antioxidant enzymes in cells, and it is easy to penetrate the blood–brain barrier. But its antagonistic effect on age‐related CIRI and potential medicinal value are still unknown. Nuclear factor erythroid 2‐related factor 2 (Nrf2) is the most important transcription factor regulating the expression of antioxidant proteins in the body. This study first compared the pathological differences of the Nrf2 system in CIRI between 2‐month‐old and 12‐month‐old Sprague–Dawley (SD) rats. Subsequently, EC was administered to 12‐month‐old rat models of middle cerebral artery occlusion and reperfusion (MCAO/R) and senescent SH‐SY5Y cell models subjected to oxygen glucose deprivation/reoxygenation (OGD/R). EC treatment improved cerebral morphology and function; increased p‐Nrf2, heme oxygenase‐1 (HO‐1), superoxide dismutase (SOD), and glutathione (GSH) expression; reduced infarct volume; and neuronal apoptosis in senescent rats. Moreover, EC enhanced cellular activity and the expression of p‐Nrf2, HO‐1, and quinone oxidoreductase‐1 (NQO‐1) while decreasing ROS and malondialdehyde (MDA) levels and mitigating apoptosis in senescent SH‐SY5Y cells. These effects were reversed upon si‐Nrf2. In sum, we confirm that EC exerts neuroprotective effects by upregulating Nrf2/ARE and reducing oxidative stress, suggesting that EC may be a promising drug for the treatment of senile cerebral apoplexy. This study also provides a scientific basis for the development and selection of new drugs for ischemic stroke in elderly patients.

## Graphical abstract

Epicatechin promotes the phosphorylation of Nrf2 into p‐Nrf2, thereby increasing HO‐1, SOD, and GSH expression and decreasing ROS and MDA. Based on the modulation of the Nrf2/HO‐1 pathway, EC mitigates oxidative stress‐induced ROS accumulation and associated oxidative damage, thus alleviating CIRI.

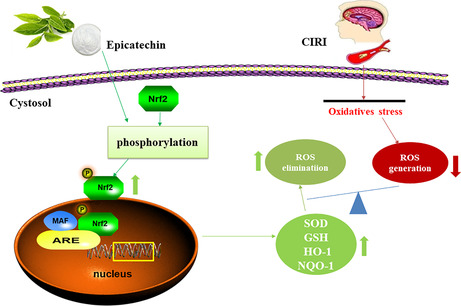

## 1 INTRODUCTION

Cerebral ischemia–reperfusion injury (CIRI) often ensues following the restoration of blood flow during stroke treatment (Orellana‐Urzúa et al., 2020). Among various physiological mechanisms, oxidative stress (OS) assumes a pivotal role in the pathophysiology of CIRI, as excessive reactive oxygen species (ROS) generation can precipitate severe brain damage (Kabir et al., 2022).

Age emerges as a significant factor influencing stroke incidence, with individuals over 65 years facing elevated mortality rates, diminished quality of life, and heightened financial burdens. Despite the reported benefits of antioxidants in clinical settings, their efficacy in treating CIRI, particularly in the elderly, remains limited. Studies indicate heightened oxidative stress in older organisms, resulting in weakened ischemia–reperfusion resistance and exacerbated brain damage (Joundi et al., 2021; Wang et al., 2022). Hence, age‐specific investigations are imperative to grasp declining endogenic antioxidative functions and explore promising therapeutics using aging models (Kabir et al., 2021).

Nuclear factor erythroid 2‐related factor 2 (Nrf2), a redox‐sensitive transcription factor, orchestrates the generation of various antioxidative proteins crucial for combating OS (Ma, 2013). However, Nrf2 expression wanes in the aging brain, potentially exacerbating ROS levels during CIRI in the elderly (Silva‐Palacios et al., 2018). Identifying natural agents modulating Nrf2 function to bolster aging nerve resistance against OS damage becomes imperative.

In our previous study, we demonstrated that epicatechin (EC) can upregulate Nrf2 expression, thus ameliorating neuronal damage caused by middle cerebral artery occlusion (MCAO) at 2 months of age. To add to our findings, we investigated the potential of EC to upregulate Nrf2 expression for alleviating CIRI in aging rats subjected to MCAO/R and senescent SH‐SY5Y cells subjected to oxygen glucose deprivation/reoxygenation (OGD/R) in vitro.

In our previous study, we demonstrated that EC can upregulate Nrf2 expression and mitigate neuronal damage post‐MCAO in younger rats. We extended our investigation to evaluate the potential of EC in upregulating Nrf2 expression to alleviate CIRI in aging rats subjected to MCAO/R and senescent SH‐SY5Y cells under OGD/R conditions in vitro.

## 2 MATERIALS AND METHODS

### 2.1 Main chemicals and reagents

Epicatechin (IE0120, Solarbio, Beijing), Nissl stain solution (Solarbio, Beijing; MDA, GSH), SOD, and bicinchoninic acid (BCA) kits were used assay kits (S0131S, S0053, S0088; Beyotime Biotechnology, Beijing); HO‐1 (Abcam, USA, ab68477), NQO‐1 (ab80588, Abcam), p‐Nrf2 (PA5‐67520, Thermo Fisher, USA), glyceraldehyde‐3‐phosphate dehydrogenase (GAPDH; 1:1000, Cell Signaling Technologies, USA, #5174), and β‐tubulin (1:1000, Cell Signaling Technologies); lactate dehydrogenase (LDH) and ROS Assay Kit (A020‐2‐1, ES004‐1‐1, Nanjing Jiancheng, China); fetal bovine serum (FBS; GIBCO 10100147, USA); and Deferoxamine mesylate (DFO, Apex BIO, Cat. No: B6068, USA) were also purchased.

### 2.2 Experimental animals and middle cerebral ischemia reperfusion modeling

Male Sprague–Dawley rats were provided by the Animal Center of Guangxi Medical University (Nanning, China), and the animal procedures were approved by the Animal Ethics Committee of Guangxi Medical University (No. 202009103). The MCAO method was used to establish a focal cerebral ischemia–reperfusion model. Ligation and insertion of pins were not performed in Sham groups. Firstly, four groups are divided to compare the differences between 2‐ and 12‐month‐old rats after MCAO/R. Then, five groups were randomly divided into 12‐month‐old rats: model group (MCAO group); positive control group (edaravone group); and three EC‐treated groups. Following this, 0.9% saline (NS) and different doses of EC (5, 10, and 20 mg/kg; Huang et al., 2021) and edaravone (3 mg/kg) were administered via the femoral vein immediately after resuscitation. 2% isoflurane (65922154, Shanghai Yuyan Instruments Co., Ltd) inhalation anesthesia was given in advance of performing surgery and before sacrifice to decrease the pain in rats.

### 2.3 Neurological and morphological evaluation

The Longa‐5‐point scale is a frequently used method for neurological and morphological evaluation (Longa et al., 1989). The damage to nerve function is more severe when the scores are high. The volume of cerebral infarction in rats is commonly measured by diphenyltetrazolium chloride (TTC) staining. At 36 h after ischemia–reperfusion, quickly take the rat brain tissues frozen in the refrigerator at −20°C for about 15 min. After the removal of parts that were not required, such as the olfactory bulb and brain stem, they were cut into 2‐mm‐thick brain slices, incubated in a 2% TTC solution at 37°C for 15 min, turned once every 5 min, and subsequently taken out and immersed in a 10% formalin solution overnight. Finally, we imaged and computed the cerebral infarction using the Image J software.

### 2.4 Histological examination and immunohistochemical staining

After being anesthetized with deeply inhaled 2% isoflurane, rats underwent intracardiac perfusion and in situ perfusion fixation. Brain samples were collected and fixed overnight, routinely dehydrated, and embedded in paraffin, as described previously (Hu et al., 2022).

TdT‐mediated dUTP‐biotin nick end labeling (TUNEL) staining procedures were primarily based on instructions provided in the TUNEL detection kits (Beyotime biotechnology). Following this, the slices were observed under a pathological microscope. Five different visual fields were randomly selected at 40 magnifications, and the apoptosis index was calculated.

### 2.5 MDA, LDH, SOD, GSH, and ROS assays

The activities of MDA, LDH, SOD, and GSH in rat brain tissue and SH‐SY5Y cells were detected according to the instructions of the assay kits. ROS production was measured by diacetate fluorescent probe (DCFH‐DA, USA).

### 2.6 Immunofluorescence and Western blot detection

Protein expressions were assessed by immunofluorescence staining. First, antigen repair was performed on the paraffin slices after dewaxing and dehydration. Second, the slices were treated overnight with primary antibodies (anti‐HO‐1 or anti‐Nrf2), followed by treatment with secondary antibodies. Third, use DAPI to counterstain. Lastly, observe under a fluorescence microscope.

The total protein from ischemic cerebral cortex tissues was extracted using the respective kits. After measuring their concentration, using sodium dodecyl sulfate‐polyacrylamide gel electrophoresis (SDS‐PAGE) segregated and transferred to polyvinylidene difluoride membranes (PVDF). They were then sealed with skim milk and treated with primary antibodies including anti‐HO‐1, anti‐p‐Nrf2, and anti‐β‐tubulin antibodies. Following this, the samples were re‐treated with secondary antibodies. The signals from the expressed proteins were measured using an enhanced chemiluminescence (ECL) kit. The relative expression level of proteins is equal to the ratio of the target protein to the internal reference β‐tubulin or GAPDH.

### 2.7 Cell culture

The OGD of senescent SH‐SY5Y cells was performed as described previously (Qiao‐tian et al., 2023). After OGD/R, the model group referred to fresh complete medium, whereas the drug administration group was treated with different EC concentrations (50, 100, and 200 μM) or edaravone (50 μM).

### 2.8 Nrf2 siRNA transfection

The OGD of senescent SH‐SY5Y cells were performed 3 times, added Opti Minimal Essential Medium (MEM) medium to prepare the small/short interfering RNA (siRNA) to a final concentration of 50 nM, mixed stand for 10 min, then incubated for 6 h. The transfected cells were treated with DMEM, EC, or edaravone for 24 h. Following this, cell survival was assessed.

### 2.9 Cell apoptosis detection

The senescent SH‐SY5Y cells were washed twice with ice‐cold PBS and then suspended in binding buffer. Then five microliters of Annexin V‐FITC was added and incubated at room temperature in the dark for 15 min, followed by the addition of 400 μL of binding buffer, and uploaded to the flow cytometer immediately. The percentage of apoptotic cells was calculated. The apoptotic cell percentage was calculated.

### 2.10 Statistical analysis

The results were expressed as mean ± SD. Prism 8.0 software was used to perform experimental data analysis. Bonferroni test was employed when data conformed to a normal distribution, and Mann–Whitney was used for non‐normal distributions. *p* < .05 was considered statistically significant.

## 3 RESULTS

### 3.1 Changes in the oxidative damage status of young and aging rats after I/R

**FIGURE 1:**
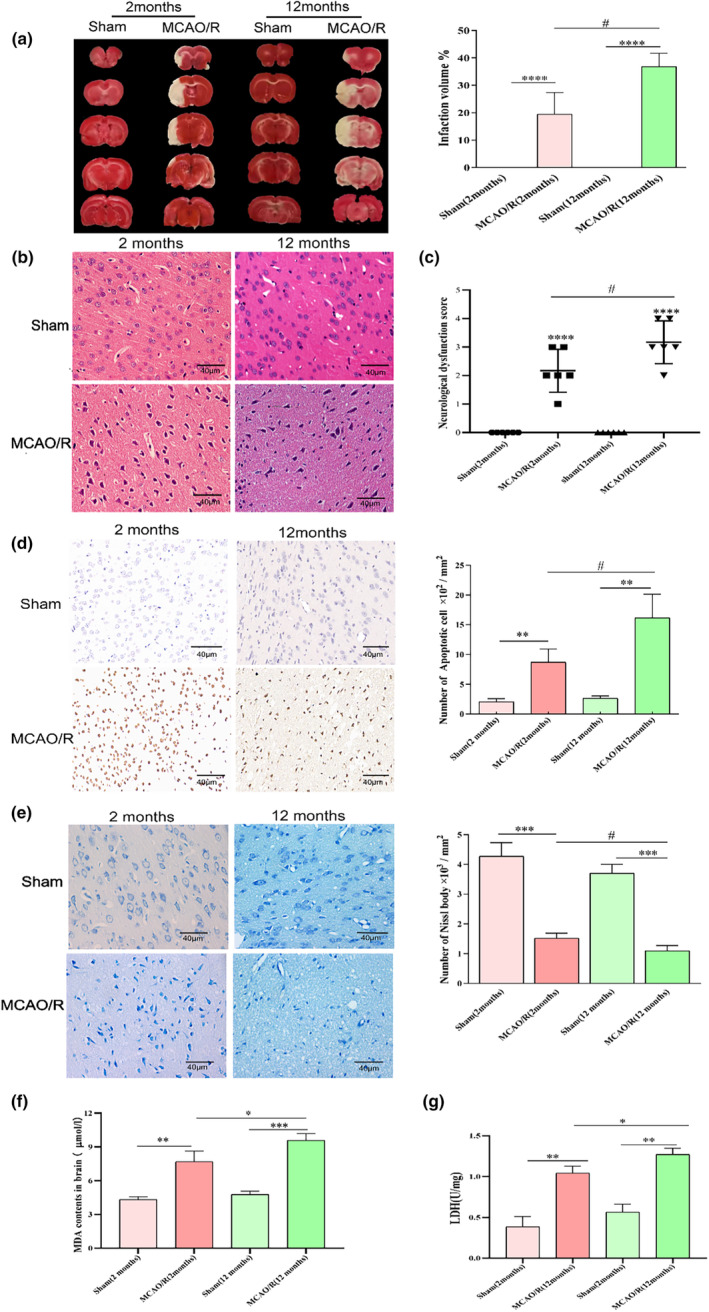
Changes in the oxidative damage status of young and aging rats after I/R. (a) Changes in coronal brain sections after staining with 2% TTC. Red‐colored regions are representative non‐ischemic, and pale regions indicate the ischemic portions. Quantitative analyses of infarct volumes were expressed as percentages (b) HE staining of the cerebral cortex. Scale bar: 40 μm. (c) Neurological deficit scores of each group. *n* = 6. (d) TUNEL staining of the cerebral cortex. Scale bar: 40 μm. (e) Nissl staining of the cerebral cortex. Scale bar: 40 μm. (f, g) MDA (f) and LDH (g) contents. All data are represented as the mean ± SEM. *p < .05, **p < .01, ***p < .005, ****p < .0001 versus the Sham group. #*p* < .05 versus the MCAO/R group.

The neurological deficit scores in each group were determined after 36 h of developing the models. No neurological deficits were observed in rats from the Sham group at 2 and 12 months of age, and rats from both MCAO/R groups showed varying degrees of elevated scores. In MCAO/R, there was more serious damage in the 12‐month group than in the 2‐month group (Figure 1a). According to the TTC staining results, no infarct focus was observed in the Sham groups, whereas the MCAO/R groups showed infarcts more or less. The white infarct volume in the 12‐month MCAO/R group was larger than that in the 2‐month‐old group. HE staining showed morphological changes in rats after CIRI. As shown in Figure 1b, there were abundant cells that were closely arranged, round, and clear, with the nucleoli positioned centrally in the Sham group. The MCAO/R groups showed a reduced number, were arranged in a dispersed manner, had irregular morphology, and some cells showed nuclear pyknosis and pathological damage. Compared with the 12‐month MCAO/R groups, the 2‐month groups showed elevated cells with regular morphology. Neurological function scores are used to assess the dysfunction in neurons after MCAO/R to some extent. As shown in Figure 1c, the MCAO/R (12‐month) groups had higher scores than the MCAO/R (2‐month) groups. This implies that neurological functions were more severely affected. Besides, TUNEL staining is used to observe apoptosis after CIRI. After TUNEL staining, the nuclei of normal cells were blue, and those of apoptotic cells were brown. The results showed that apoptotic nuclei were barely present in the Sham group, whereas they increased markedly in the MCAO/R group (Figure 1d). In the MCAO/R groups, there was a significantly higher apoptosis rate in the 12‐month group. Generally, Nissl bodies are stored in surviving neurons. As expected, the maximum number of Nissl bodies exists in the Sham group, and the MCAO/R group has the least (Figure 1e). Interestingly, the number of Nissl bodies in the 12‐month group was less than that in the 2‐month group. Both 2 and 12‐month MCAO groups showed a significant increase in MDA (Figure 1f) and LDH level (Figure 1g). However, the MDA and LDH contents were higher in the infarction penumbra in the 12‐month group than in the 2‐month group. The above findings suggested that age attenuated oxidative damage and MCAO/R‐induced neuronal death.

### 3.2 Differences in antioxidant capacity between young and aging rats after I/R

**FIGURE 2:**
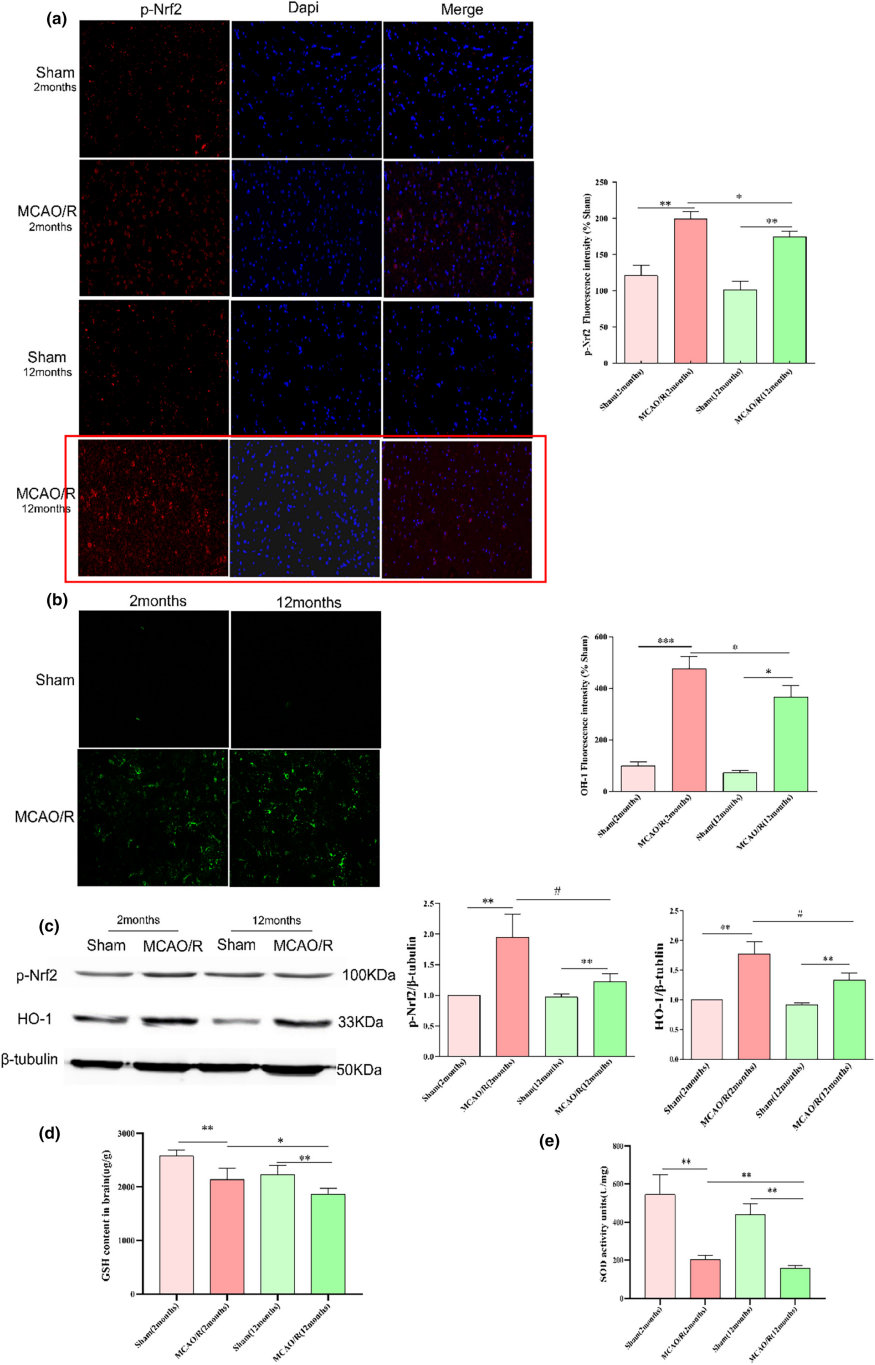
Differences in antioxidant capacity between young and aging rats after I/R. (a) immunofluorescence staining of p‐Nrf2. (b) immunofluorescence staining of HO‐1. (c) WB analysis of p‐Nrf2, HO‐1. (d, e) GSH content (d) and SOD activity (e). The data above are represented as the mean ± SEM of three times and analyzed by the Kruskal–Wallis test. **p < .01, ***p < .005 versus the Sham group. #*p* < .05 versus the MCAO/R group.

First, we investigated whether the p‐Nrf2 expression level reflects the level of antioxidant capacity. We measured the changes in the p‐Nrf2 levels in each group using immunofluorescence. After MCAO/R, p‐Nrf2 expression was significantly downregulated in the 12‐month group (Figure 2a). The same result was observed in the western blotting experiment (Figure 2c). HO‐1 expression is an important antioxidant index. HO‐1 levels decreased, accompanied by the level of p‐Nrf2 (Figure 2b). To better understand the changes in the Nrf2 pathway in 2‐ and 12‐month rats, we performed western blotting using brain tissues. The same result was verified, as shown in Figure 2c. These results were consistent with findings from previous studies, suggesting that the Nrf2 pathway is a marker for aging. As evidence from multiple independent studies often shows the association between GSH expression and the antioxidative index, we next focused on GSH expression. As shown, the GSH levels decreased after MCO/R, with a greater reduction observed in the 12‐month group than in the 2‐month model group (Figure 2d). SOD serves as a marker for antioxidative potential in the brain. We thus checked the changes in the expression of SOD. The SOD content was markedly higher in the 2‐month group than in the 12‐month model group (Figure 2e). These data suggested that Nrf2 expression is age‐associated, and along with antioxidant proteins, Nrf2 might play a functional role in physiological CIRI processes with aging.

### 3.3 EC mitigated oxidative damage in CIRI

**FIGURE 3:**
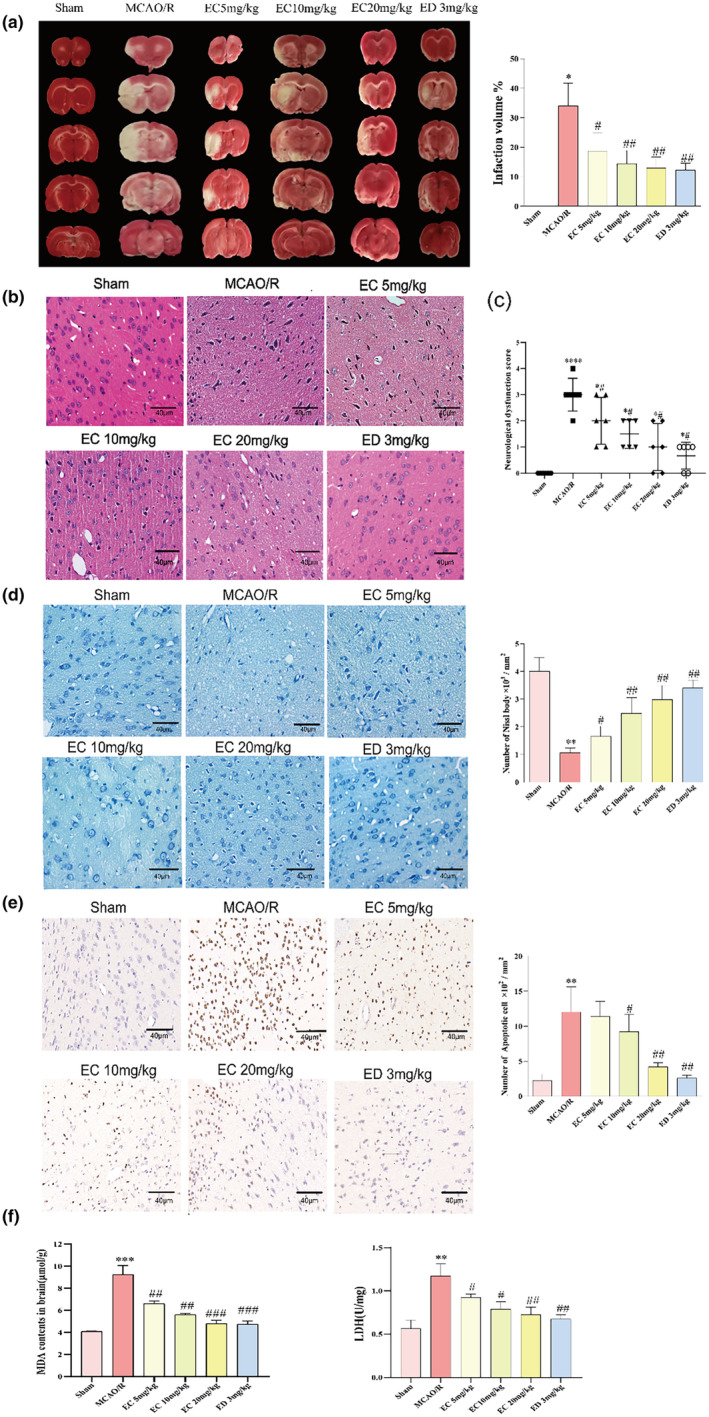
Epicatechin (EC) reduces oxidative damage in cerebral ischemia–reperfusion. (a) TTC staining and quantification of cerebral infarct volume. (b) Representative images of HE staining in the groups. Scale bar: 40 μm. (c) Neurological deficit scores of each group. (d) Nissl staining of the cerebral cortex. Scale bar: 40 μm. (e) TUNEL staining of nerve cells with different concentrations of EC. Scale bar: 40 μm. (f) MDA and LDH were detected by the assay kit. The data in the figures are represented as the mean ± SEM and analyzed by the Kruskal–Wallis test. *n* = 3, **p < .01 versus the SH group. ###*p* < .001, ##*p* < .01, and #*p* < .05 versus the MCAO/R group. The data are presented as mean + SD.

The most obvious feature of MCAO/R‐induced injury is aggravated brain damage and cerebral infarction. To assess the cerebral protective effect of EC, we evaluated neuronal damage using TTC staining. We observed that treatment with MCAO/R markedly increased the infarct volume ratio in comparison to the model group, which was reversed by EC (Figure 3a). This indicated serious neurological deficits in the MCAO/R model. HE staining showed that the number of neurons reduced considerably after MCAO/R, whereas it increased in response to EC treatment (Figure 3b). Neurological function scores were also assessed. MCAO/R showed the highest score among all groups. Whereas, EC treatment got a score close to that of Sham group (Figure 3c). Nissl staining is used to assess dysfunction in neuronal protein synthesis. Neuron loss, nuclei faint staining, swelling, and vacuolar changes were found in the MCAO/R groups. More Nissl bodies and deeper staining as a whole were showed in EC group, especially in the EC 20 mg/kg group (Figure 3d). In the brain tissues, CIRI induced apoptosis. Cells with a brown stain are apoptotic cells in TUNEL staining. Upon treatment with different doses of EC, the number of apoptotic cells increased to varying degrees compared to the MCAO/R group (Figure 3e). Based on the data described above, we further confirmed the damage at the biochemical level. MDA and LDH expression elevated after MCAO/R, and EC partially decreased it (*p* < .05; Figure 3f). These results above suggest that EC could reduce the negative impacts of oxidative damage induced by MCAO/R.

### 3.4 EC enhances the antioxidant potential of aging rats

**FIGURE 4:**
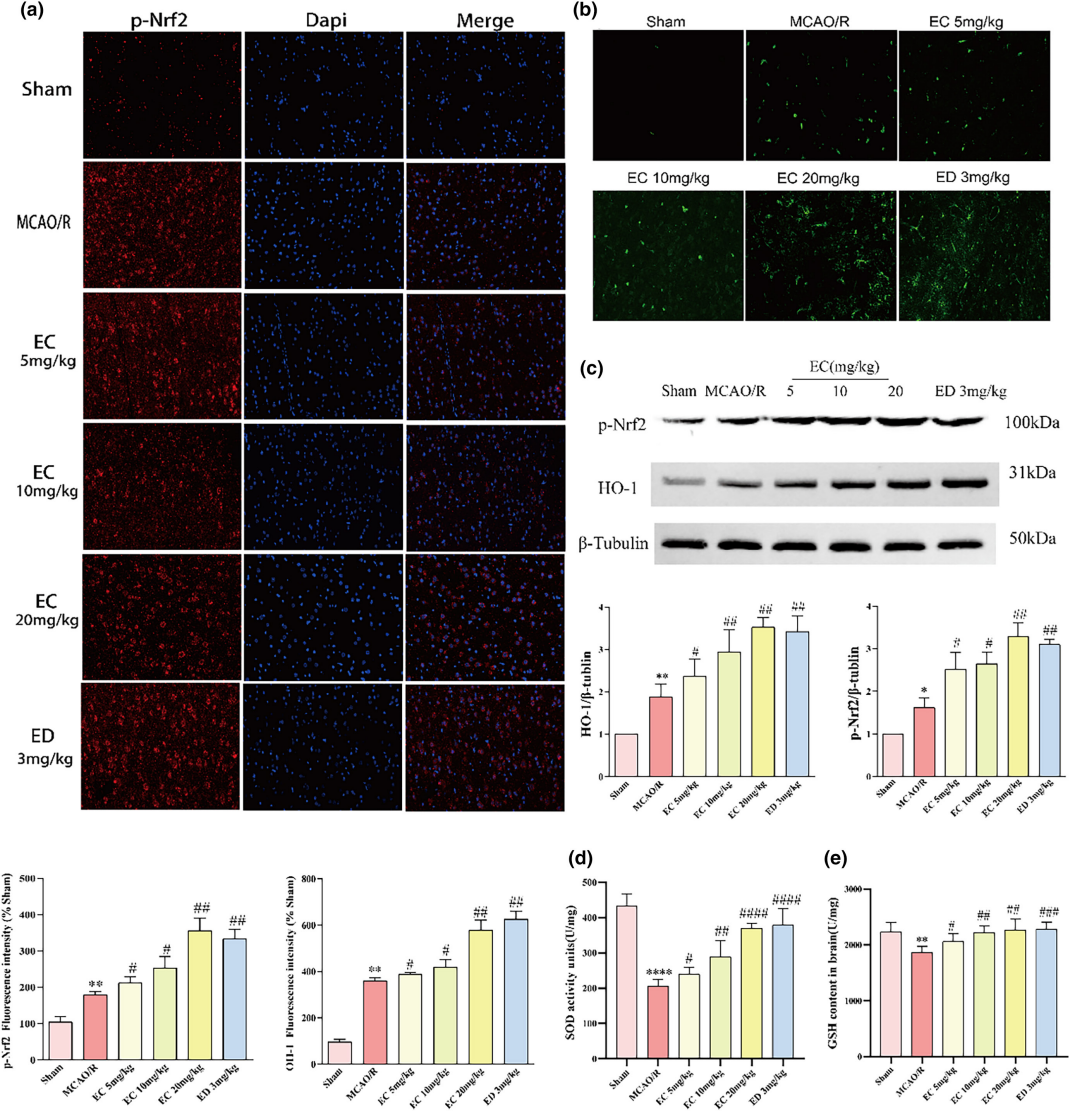
Epicatechin (EC) enhances the antioxidant potential of aging rats. (a) Dual immunofluorescence in p‐Nrf2 (red) and nuclei (blue) was visualized by Dapi staining. (b) Dual immunofluorescence in HO‐1 (green) was visualized by Dapi staining. Scale bars represent 40 μm. (c) Western blot analysis of p‐Nrf2 and HO‐1 in infarct lateral brain tissue. After densitometric analysis, β‐tubulin was used as an internal reference for relative quantification. (d, e) SOD and GSH contents were determined using their respective detection kits after I/R. Data represent the mean ± SD, *n* = 3 per group, *p < .05, **p < .01 compared with the Sham group; #*p* < .05, ##*p* < .01 compared with the MCAO/R group.

To investigate the mechanism of EC, the expression/activation of the Nrf2 pathway were assessed. Nrf2 signaling is the key endogenous antioxidant signaling cascade. Once activated, Nrf2 will separate from Keap1, which will lead to Nrf2 phosphorylation. To determine how EC exerts a neuroprotective effect, we studied the antioxidant protein p‐Nrf2. As in Figure 4a by the overlapping evaluation of the red spots of p‐Nrf2 with the green spots of the nucleus. The greater the overlapping region, the higher the p‐Nrf2 infiltration into the nucleus. We observed that the p‐Nrf2 level in the cytoplasm increased after MCAO/R, and the fluorescence intensity of p‐Nrf2 in the nucleus was significantly enhanced after the EC treatment group versus the MCAO/R group. We verified this result simultaneously using western blotting (Figure 4c). HO‐1 plays an important role in the antioxidant defense system. Green fluorescence increased with the EC dose (Figure 4b). The same result was verified in Figure 4c. The improvement of the antioxidant capacity of the organism by EC was also investigated, and GSH and SOD in fresh brain samples were measured using corresponding kits. The GSH content reduced significantly after MCAO/R. EC boosted GSH content, especially in the 20 mg/kg group (Figure 4d). The SOD activity decreased after MCAO/R, whereas EC treatment increased its activity (Figure 4e). These results suggest that EC treatment can obviously suppress oxidative stress induced by MCAO/R and facilitate the activation of the Nrf2/HO‐1 signaling pathway in vivo.

### 3.5 EC reduced oxidative injury during OGD/R in senescent SH‐SY5Y cells

**FIGURE 5:**
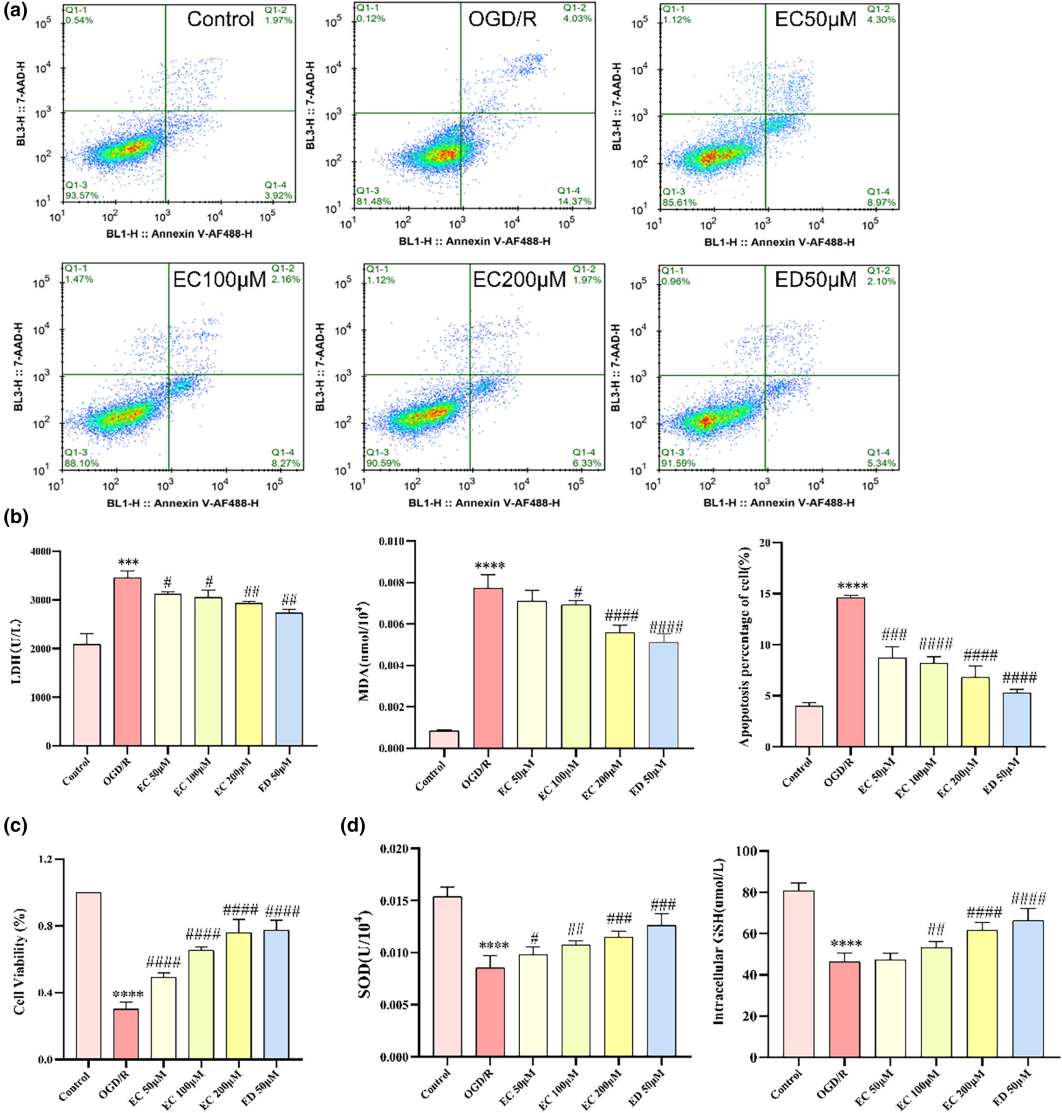
EC reduces oxidative injury during OGD/R in senescent SH‐SY5Y cells. (a) Cell survival rate of EC‐treated senescence SH‐SY5Y cells. (b) LDH and MDA contents were elevated in response to OGD/R in senescence SH‐SY5Y cells subjected to OGD/R. (c) Cell ability of EC‐treated in senescence SH‐SY5Y cells after OGD/R. (d) Effects of EC on SOD and GSH contents after OGD/R. *p < .01 versus the control group; #*p* < .05; and ##*p* < .01 versus the OGD/R group.

In the previous part of the experiment, we observed that EC reduced oxidative stress in vivo. Thus, we speculated whether EC could promote an anti‐oxidative effect in vitro. Senescent cells in the nervous system accelerate apoptosis with aging; previous studies have shown the induction of apoptosis by OGD/R. We measured the apoptosis‐related parameters to determine whether preconditioning with EC can prevent OGD/R‐induced apoptosis (Figure 5a). To confirm these observations, we also evaluated the LDH content using a kit (Figure 5b). To evaluate lipid peroxidation, we tested MDA (Figure 5b). The percentage of MDA levels increased with OGD/R. Cell viability was assessed using a CCK8 assay kit. EC treatment significantly restored cell viability, which was suppressed by OGD/R, in senescent SH‐SY5Y cells (Figure 5c). As illustrated in Figure 5d, the levels of SOD were significantly increased as well as GSH levels in the OGD/R group in response to oxidative stress. Conversely, EC attenuated the levels of LDH as well as MDA. Together, these findings indicated that the absence of EC accelerates susceptibility to OGD/R injury.

### 3.6 si‐Nrf2 reversed the protective effects of EC

**FIGURE 6:**
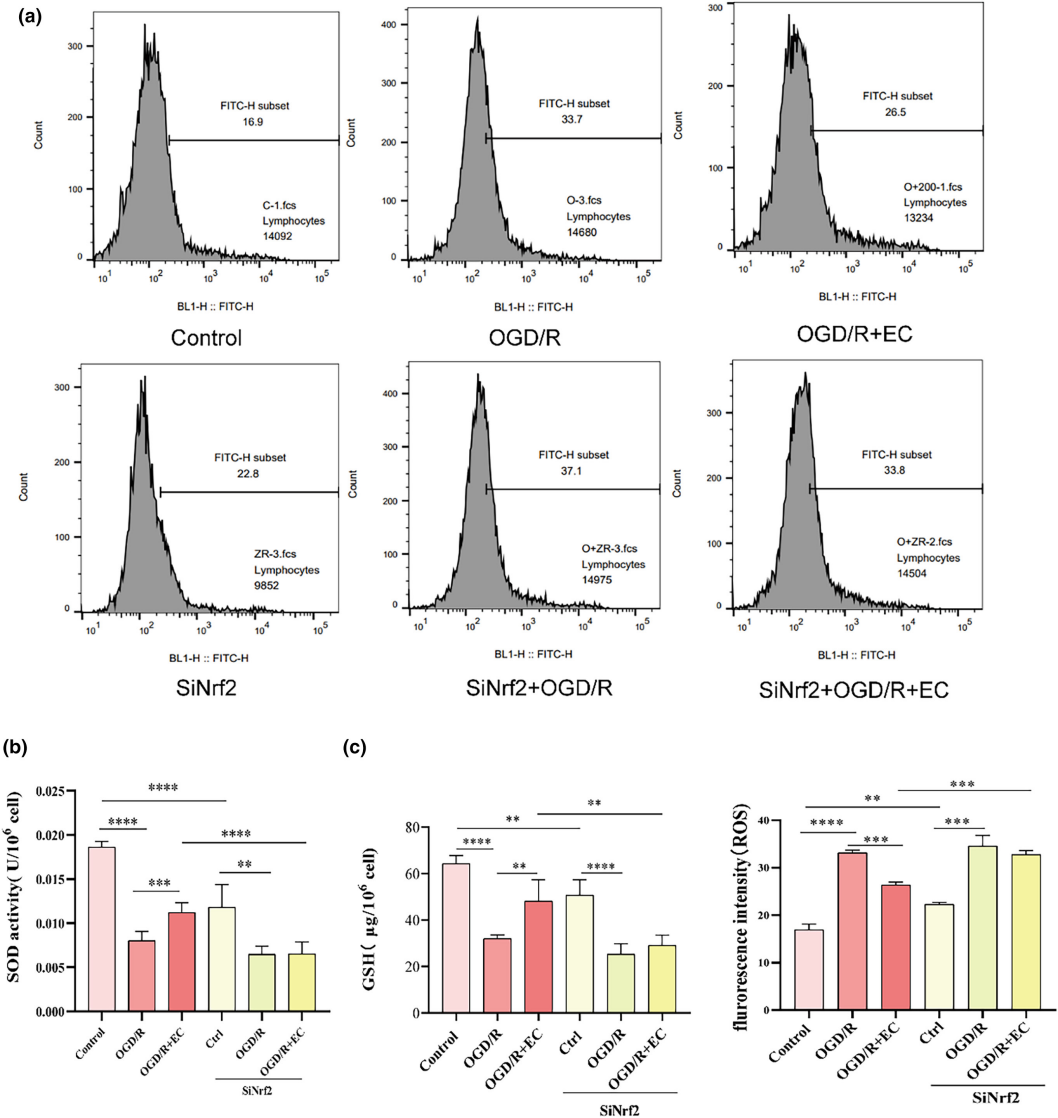
Si‐Nrf2 reversed the protective effects of epicatechin (EC). (a) ROS was detected via flow cytometer after transfection of SiNrf2 and treatment with EC. (b, c) The levels of SOD (b) and SGH (c) were determined using their respective detection kits after transfection of SiNrf2. *p < .05, **p < .01, ****p < .001.

Next, we explored whether Nrf2 was involved in OGD/R‐induced oxidative stress. OGD/R stimulation in neuronal cells induced profound ROS generation and oxidative stress, causing cell death and apoptosis. The findings of the flow cytometry assay revealed that more ROS generation was induced in OGD/R than in the control group. EC obviously decreased ROS generation. In contrast, in the si‐Nrf2 + OGD/R group, we did not see a significant change compared with the si‐Nrf2 + OGD/R + EC group (Figure 6a).

To explore whether Nrf2 was involved in OGD/R‐induced antioxidant capacity, the content of SOD was measured in senescent SH‐SY5Y cells because SOD generation increased and subsequent oxidative activation occurred in EC‐treated cells. Next, we determined whether SOD played a role in the results observed in the si‐Nrf2 group. EC could exert the neuroprotective and neurorestorative roles. However, these findings were not observed in the si‐Nrf2 group (Figure 6b). This suggests that EC enhances antioxidant capacity via Nrf2 in OGD/R. The concentration of GSH was assessed in senescent SH‐SY5Y cells. The GSH levels were lower in ODG‐treated SH‐SY5Y cells but increased in response to EC treatment (Figure 6c). This indicated that EC effectively promoted the antioxidant potential in OGD‐treated cells. However, these alterations were reversed upon si‐Nrf2 introduction. These findings support the fact that EC has neuroprotective effects that are potentially mediated by Nrf2. Conversely, EC + Nrf2 group was not less in preconditioning SH‐SY5Y cells (Figure 6c). These results revealed that EC reduced OGD/R‐induced oxidative injury in SH‐SY5Y cells by partly relying on the upregulation of the Nrf2 pathway.

## 4 DISSCUSSION

While CIRI involves various mechanisms, oxidative stress stands out as a primary contributor to tissue damage (Orellana‐Urzúa et al., 2020). The escalating incidence of stroke poses a substantial societal burden as the world grapples with an aging population. Elderly individuals face more severe brain damage and encounter greater challenges in recovery due to heightened oxidative stress levels (Lonati et al., 2022). However, there remains a dearth of research on drugs aimed at mitigating CIRI in the elderly. Thus, understanding the manifestations of antioxidant pathways with aging and utilizing aging models to investigate antioxidant drugs are imperative (Tagde et al., 2021). Despite previous reports on the effects of EC in CIRI (Leonardo et al., 2013), its exploration in aging models remains limited. Our study demonstrates that EC can alleviate senile CIRI by enhancing the antioxidative function of the Nrf2 system.

Initially, we compared CIRI between 2‐month‐old and 12‐month‐old Sprague–Dawley (SD) rats, revealing an attenuation of Nrf2 function with aging alongside increased oxidative stress (OS) levels and heightened CIRI severity. Consequently, rats in the 12‐month group exhibited more severe structural and neurological damage in the brain. Aging animals are predisposed to earlier blood–brain barrier disruption, larger infarct volumes, increased neuronal degeneration, and worse neurological outcomes in ischemic stroke compared to their younger counterparts (Bayliak et al., 2021).

In the subsequent phase, we investigated the anti‐CIRI effects of EC in 12‐month‐old rats. EC, a plant polyphenol abundant in the human diet, possesses two aromatic rings and an oxygenated heterocycle (Shimura et al., 2019), along with multiple phenolic hydroxyl groups capable of scavenging ROS both directly and indirectly (Grewal et al., 2021). These distinctive pharmacological properties set EC apart from other flavanols. Moreover, studies have demonstrated EC can penetrate the blood–brain barrier, along with its neuroprotective effects (Wu et al., 2012). To assess the impact of EC on oxidative homeostasis, we evaluated GSH and MDA contents as well as SOD activity in brain tissues. We observed a gradual increase in SOD activity and GSH content in the EC group with increasing EC concentration, accompanied by a decrease in MDA content, which is associated with improved Nrf2 function in response to EC treatment. In a reperfusion experiment conducted 60 minutes after MCAO, Nrf2 knockout mice exhibited more pronounced neurological deficits and significantly larger infarct areas than wild‐type mice (Liu et al., 2019; Lonati et al., 2022). Conversely, Nrf2 upregulation resulted in a reduction in brain injury (Sun et al., 2020). The experimental findings in the present study are consistent with these studies.

To investigate the effect of EC in vitro, an oxidative test was conducted on senescent SH‐SY5Y cells treated with varying doses of EC in the OGD/R model. The results indicate that EC improves the endogenous antioxidant capacity of senescent cells by upregulating GSH levels and SOD activity, reducing MDA production, and decreasing apoptosis, thus mitigating OGD/R injury. Previous studies have reported similar observations, with EC having been shown to reduce cellular OGD/R‐induced apoptosis in neuronal cells by enhancing mitochondrial function (Ramirez‐Sanchez et al., 2014).

To investigate whether EC enhances endogenous antioxidant function by regulating Nrf2 expression, we conducted a Nrf2 silencing experiment and observed changes in SOD and GSH, downstream antioxidants regulated by Nrf2, which induce the expression of SOD and GSH synthases. Glutathione biosynthesis is tightly controlled by the rate‐limiting enzyme glutamate‐cysteine, transcriptionally regulated by Nrf2 (Zhang et al., 2020). In this study, we found that SOD and GSH levels in the si‐Nrf2 group were lower than in the non‐si‐Nrf2 group, and both were further reduced in response to OGD/R treatment. However, EC significantly upregulated the expression of SOD and GSH in the non‐si‐Nrf2 group, thereby preventing OGD/R‐induced MDA and ROS production, whereas this effect was absent in the si‐Nrf2 group. These results suggest that EC mitigates oxidative stress‐induced ROS accumulation and associated oxidative damage. Nrf2 expression is impaired with age, leading organisms into a chronic oxidative state (Ungvari et al., 2019). Mice become more susceptible to oxidative damage when Nrf2 is knocked out (Song et al., 2020). Si‐Nrf2 suppresses the antioxidant function of Nrf2 (Pellegrini et al., 2017). Our findings demonstrate that Nrf2 silencing decreased antioxidant potential and increased apoptosis and ROS production, possibly owing to impairment of the Nrf2/HO‐1 pathway. Mechanistically, EC has been implicated in the regulation of Nrf2 phosphorylation via p‐Nrf2 expression and nuclear transportation, which in turn elevates HO‐1 and NQO‐1 protein levels and reduces ROS accumulation.

EC has been shown to improve the functional decline of Nrf2 associated with aging, thereby mitigating oxidative stress and apoptosis during CIRI. Our results suggest that this plant‐derived compound may hold therapeutic potential in CIRI. However, our study design had certain limitations, as we only utilized a 12‐month‐old Sprague–Dawley rat model of CIRI injury in the animal experiments. In future investigations, we intend to include observations of the cerebral protective effects of EC in rats at 18 and 24 months of age.

## 5 CONCLUSIONS

**FIGURE 7:**
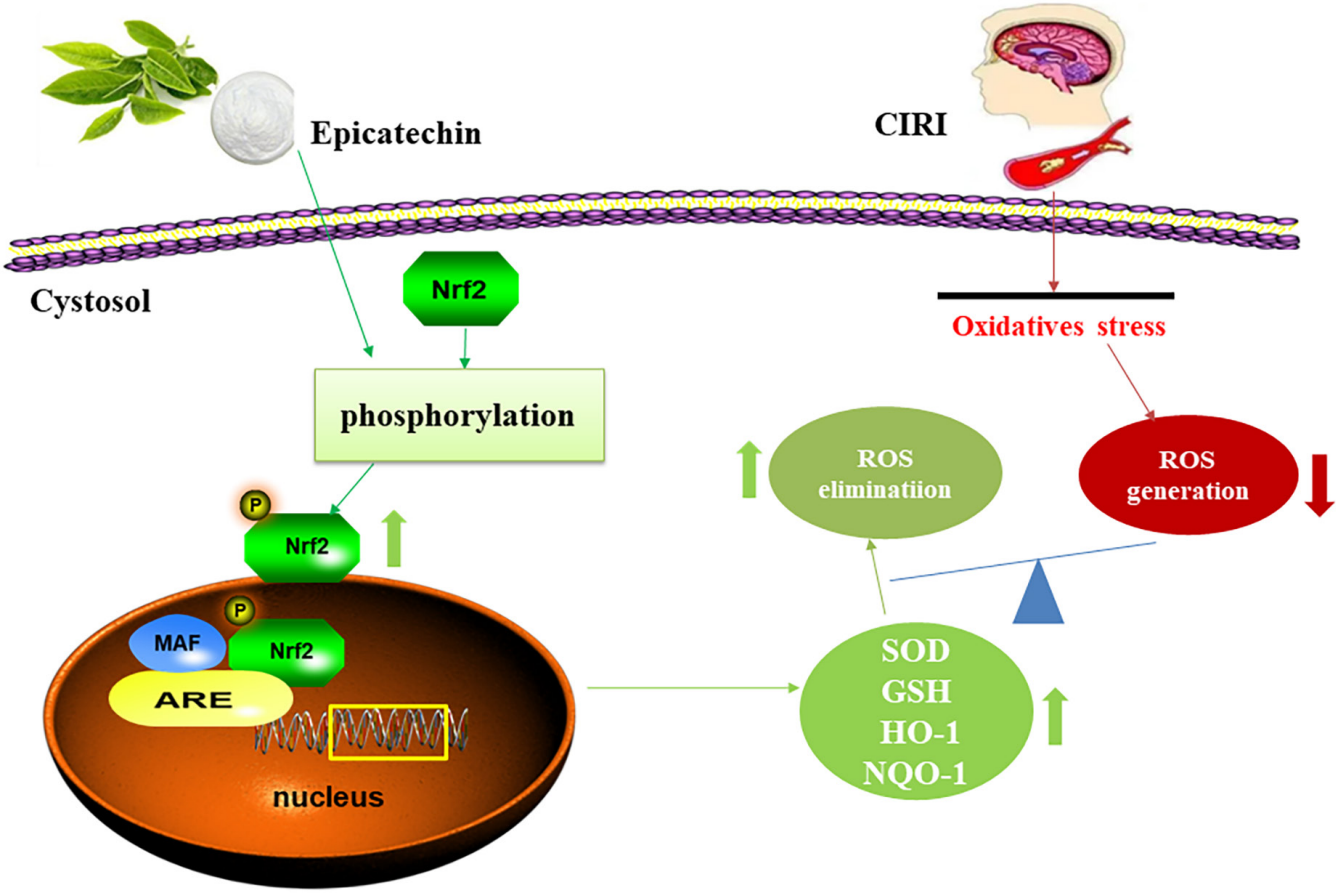
Mechanism of epicatechin (EC) antagonism to CIRI via enhanced Nrf2/HO‐1 pathway.

In summary, we investigated the neuroprotective effects and underlying mechanisms of EC in a 12‐month‐old MCAO rat model and an OGD/R model of senescent cells. Our findings shed light on the mechanism of action of EC in reducing oxidative stress‐induced damage through modulating the Nrf2/HO‐1 pathway (Figure 7), indicating that EC has potential as a therapeutic target for age‐related stroke.

## AUTHOR CONTRIBUTIONS

**Xiangzhen Zhuge:** Data curation (equal). **Deli Li:** Investigation (equal). **Menghua Chen:** Software (equal). **Wanxiang Hu:** Methodology (equal).

## FUNDING INFORMATION

This work was supported by the National Natural Science Foundation of China (No. 82160372, 81860333), Natural Science Foundation of Guangxi (No. 2018GXNSFAA050153), 2024 Middle/Young aged Teachers’ Research Ability Improvement Project of Guangxi Higher Education (2024KY0121), and Youth Research Foundation of Guangxi Medical University (GXMUYSF202127).

## CONFLICT OF INTEREST

The authors declare that they have no competing interests.

## Data Availability

Not applicable.
